# Study protocol on comparative effectiveness of mindfulness meditation and qigong on psychophysiological outcomes for patients with colorectal cancer: a randomized controlled trial

**DOI:** 10.1186/s12906-017-1898-6

**Published:** 2017-08-08

**Authors:** Rainbow T. H. Ho, Adrian H. Y. Wan, Jessie S. M. Chan, S. M. Ng, K. F. Chung, Cecilia L. W. Chan

**Affiliations:** 1 Centre on Behavioral Health, The University of Hong Kong, 2/F, The Hong Kong Jockey Club Building for Interdisciplinary Research, 5 Sassoon Road, Pokfulam, Hong Kong; 2 Department of Social Work and Social Administration, The University of Hong Kong, Room 534, Jockey Club Tower, The Centennial Campus, Hong Kong, China; 3 Department of Psychiatry, The University of Hong Kong, Queen Mary Hospital, 102 Pokfulam Road, Hong Kong, China

**Keywords:** Mindfulness, Qigong, Baduanjin, Colorectal cancer, Randomized controlled trial (RCT), Salivary cortisol

## Abstract

**Background:**

Colorectal cancer imposes threats to patients’ well-being. Although most physical symptoms can be managed by medication, psychosocial stressors may complicate survival and hamper quality of life. Mindfulness and Qigong, two kinds of mind-body exercise rooted in Eastern health philosophy, has been found effective in symptoms management, improving mental health, and reducing stress. With these potential benefits, a randomized controlled trial (RCT) is planned to investigate the comparative effectiveness of mindfulness and Baduanjin intervention on the bio-psychosocial wellbeing of people with colorectal cancer.

**Methods/ design:**

A 3-arm RCT with waitlist control design will be used in this study. One hundred eighty-nine participants will be randomized into (i) Mindfulness, (ii) Baduanjin, or (iii) waitlist control groups. Participants in both the Baduanjin and mindfulness groups will receive 8-weeks of specific intervention. All three groups will undergo four assessment phases: (i) at baseline, (ii) at 4-week, (iii) at 8-week (post-intervention), and 6-month post-intervention (maintenance). All participants will be assessed in terms of cancer-related symptoms and symptom distress, mental health status, quality of life, stress level based on physiological marker.

**Discussion:**

Based on prior research studies, participants in both the mindfulness and Baduanjn intervention group are expected to have better symptoms management, lower stress level, better mental health, and higher level of quality of life than the control group. This study contributes to better understanding on the common and unique effectiveness of mindfulness and Baduanjin qigong, as such patients and qualified healthcare professionals can select or provide practices which will produce maximum benefits, satisfaction, adherence, and sustainability.

**Trial registration:**

The trial has been registered in the Clinical Trials Centre of the University of Hong Kong (HKCTR-2198) on 08 March 2017.

## Background

Colorectal cancer imposes threats to patients’ well-being. Although most physical symptoms can be managed by medication, psychosocial stressors may complicate survival and hamper quality of life [1]. People with colorectal cancer, especially those with colostomy, often experience psychosocial distresses such as depression, fear of relapse, altered self-image, and loss of social functioning and connections [2]. A recent study proposed reciprocity between physical symptoms and psychosocial stresses in cancer patients [3]. Studies in psycho-immunology have suggested that many cancer patients also have depressive symptoms and experience sleep disturbance, the latter of which can be a risk factor for depression and disease progression among cancer patients [4]. In addition, a survival study highlighted the connection between survival and depression, anxiety, and quality of life in patients with colorectal cancer [5]. Given the potential effects of psychosocial well-being on colorectal cancer survivorship, there is a need for psychosocial support that targets the reciprocal mind-body relationship in these patients.

The past two decades have witnessed the proliferation of rigorous empirical testing of cancer-related psychosocial interventions, such as cognitive-behavioral therapies, psycho-educational interventions, social support groups, and mindfulness-based programs [6–10]. Mind-body practices, such as relaxation training, meditation, qigong (also known as Chi-Gong), yoga, taiji (also known as tai chi), and biofeedback have been found to improve functioning; reduce treatment-related nausea, vomiting, and physical pain; and improve mood and quality of life [11, 12]. Mindfulness and qigong practice in particular have become increasingly popular because they are coupled with marked evidence pertaining to safety, feasibility, and effectiveness on various aspects of health in cancer patients.

### Mindfulness and qigong as mind-body practices for cancer

The practice of mindfulness focuses on the development of awareness of emotions and sensations, the nurturing of a nonjudgmental mindset, and self-acceptance through practice [13]. The here-and-now orientation of mindfulness has been found to be effective in moderating cancer-related stressors, such as attribution of cancer, regrets about past life decisions, suffering of pain, loss, and grief regarding one’s own impending death [9, 10]. In a randomized, waitlist-controlled trial, the participants in the mindful meditation-based program resulted in less mood disturbance, tension, stress, and depressive symptoms and improved sleep and gastrointestinal symptoms after the intervention and at the 6-month follow-up visit, when compared with the control group [14]. Mindfulness meditation was also found to be effective in altering cortisol and immune patterns consistent with lower stress with sustainable effects at the 1-year follow-up visit [15]. Local studies on the application of mindfulness in a cancer population are rare, but the results of studies on the effects of mindfulness on chronic pain, psychiatric symptoms, and functioning are promising [16, 17].

Similar to mindfulness practice in its emphasis on practicing breath-centering techniques and developing awareness of emotions and sensations, qigong is a mild form of muscular activity accompanied by introspective and proprioceptive focus and an awareness of the flow of intrinsic energy, which is essential in the pursuit of health and well-being [18, 19]. Of all types of qigong practices, Baduanjin is the most popular. The practice of Baduanjin comprises eight movements and requires relatively little cognitive and physical effort [20], which makes it a popular form of exercise for people who are physically weak, including older adults and cancer patients during rehabilitation [21, 22]. In support of its positive effects in cancer patients, a systematic review of 13 randomized controlled trials concluded that the practice of qigong improves cancer-specific quality of life, fatigue, and immune functioning; alters cortisol levels [23]; and alleviates sleep disturbance [24], which is also a common issue among cancer patients.

Although they share the common components of breath-centering practice, mindfulness and qigong represent two very distinct approaches to mind-body practices. Mindfulness meditation is a technique used to cultivate the mind’s ability to influence bodily functions and symptoms by means of focused-awareness training [12], whereas qigong capitalizes on movement and attention to foster the mind-body connection via the physical body [20, 25, 26]. The foregoing review suggests how most studies on mindfulness and qigong have examined their effects independently, which calls for comparative intervention research to elucidate how such similar, yet contrasting, approaches to mind-body practices compare in terms of their psychosocial and physiological benefits and patient compliance.

### Diurnal Cortisol rhythm and cancer

There is a physio-psychological basis for the prognosis of colorectal cancer. Studies have indicated that dysfunction of the HPA axis is associated with tumor characteristics and survival in these patients [27–29]. Salivary cortisol is a neuroendocrine indicator of the HPA axis, which is an established and reliable physiological marker for stress-related improvements after mindfulness and qigong practices [30, 31]. Understanding the changes in cortisol profiles after mindfulness and qigong practices can further elucidate the link between mind-body practices and patients’ total well-being.

### Study design

To explore the comparative effectiveness of mindfulness meditation and Baduanjin qigong practice, this study will adopt a three-armed randomized-controlled study design with two experimental groups and a waitlist control group.

### Research objectives and study hypotheses

The first main objective of this study is to independently examine the effectiveness of mindfulness meditation and Baduanjin qigong in Chinese patients with colorectal cancer compared with controls regarding cancer-related symptoms and symptom distress, mental health, quality of life, biomarkers, and mindfulness level. The other main objective is to examine the similarities and differential effectiveness of a movement-based (Baduanjin qigong) and a mind-based (mindfulness meditation) practice on the preceding outcomes, the magnitude and pace of change, and extended compliance throughout the 8-month study. The final objective is to examine the relationships among the physical, psychological, and psychophysical variables and their effects on mind and body. This study envisions that advancing relationships change over time.

To achieve the aforementioned research objectives, the following hypotheses are proposed.

1. Baduanjin qigong is more effective than no-intervention control in (i) reducing cancer-related symptoms and symptom distress, (ii) improving mental health, (iii) enhancing quality of life, (iv) positively altering biomarkers, and (v) enhancing mindfulness levels;
2. Mindfulness meditation is more effective than no-intervention control in similar areas and directionality as in the preceding (i)-(v);
3. Similarities and differences exist between mindfulness meditation and Baduanjin qigong in (i) the type of outcome variables, (ii) the magnitude of change, (iii) the time when significant outcomes emerge, and (iv) the frequency and duration of self-practice and long-term compliance; and
4. The associations among physical and psychological variables and psychophysiological biomarkers and the changes of such associations across the time points are exploratory.

## Methods

### Study setting

This study will adopt a three-armed randomized-controlled trial design with a waitlist control group. Eligible participants will be recruited from the community via networks of colorectal cancer self-help groups and local cancer support networks.

### Eligibility criteria

Chinese-speaking patients 18 years of age or older with a diagnosis of primary colorectal cancer of any stage between 0 and III and an expected survival duration of 12 months or longer who completed their main cancer treatment within the past 6 months to 5 years will be invited to participate in the trial. Those who regularly practice Baduanjin, mindfulness meditation, or other forms of qigong, tai chi, meditation, or yoga once a week or more during the past 6 months will be excluded. Those with severe cachexia, dizziness, bone pain, nausea, significant orthopedic problem, or other contraindication to mild to moderate physical exertion, or a diagnosis of a major medical or psychiatric disorder other than cancer, will also be excluded, as will patients with recurrent colorectal cancer or other cancers.

### Participants, interventions, and outcomes

Both intervention groups will consist of eight weekly sessions (90-min each), with a total of 12 contact hours. Interventions will be held in groups of about 10 participants led by two trainers qualified in the respective intervention form, one of whom will also be a mental health professional.

- *The Baduanjin qigong intervention* will consist of eight sequential forms of movements practiced with guidance on rhythmic breathing and mindful awareness. Each cycle of the eight movements requires 10 to 15 min to complete. Four cycles will be practiced in each session, with short breaks arranged between them. In addition to the in-session practices, the participants will be encouraged to practice Baduanjin qigong for at least 20 min each day and to keep a record of their daily practice in a log book.
- *The mindfulness meditation intervention* is based on the core components of the established Mindfulness-Based Stress Reduction and Mindful Self-Compassion programs with adaptation for local practice. The focus of the group will be to nurture mindful awareness, acceptance, and self-compassion. The group sessions will be composed of a combination of mindfulness-based practice, relaxation, and yoga exercise. The basic philosophy of this intervention is to emphasize the nurturing of self-kindness and a detached attitude towards interpersonal connectedness and letting go of self-criticism, the need for control, and defensive separation in times of adversity. Adjuvant to the in-session practices, the participants will be recommended to practice the mindfulness techniques for at least 20 min each day and to keep a record of their practice.
- The waitlist control group members will continue with routine care and can join either the Baduanjin or mindfulness intervention after the 6-month post-intervention assessment.

#### Sample size

Overall, 189 participants will be required to achieve the study objectives. To achieve a statistical power of 0.8 with a medium effect size (f2 = 0.25) and a significance level of 0.05 in repeated-measures multivariate analysis of variance under the proposed three-group, four-time-point design, a sample size of 126 will be needed. Assuming an attrition rate of about 33.5%, based on prior trials on cancer patients using qigong [32], a total of 189 participants (63 per arm) will be required.

#### Recruitment strategy

**Fig. 1:**
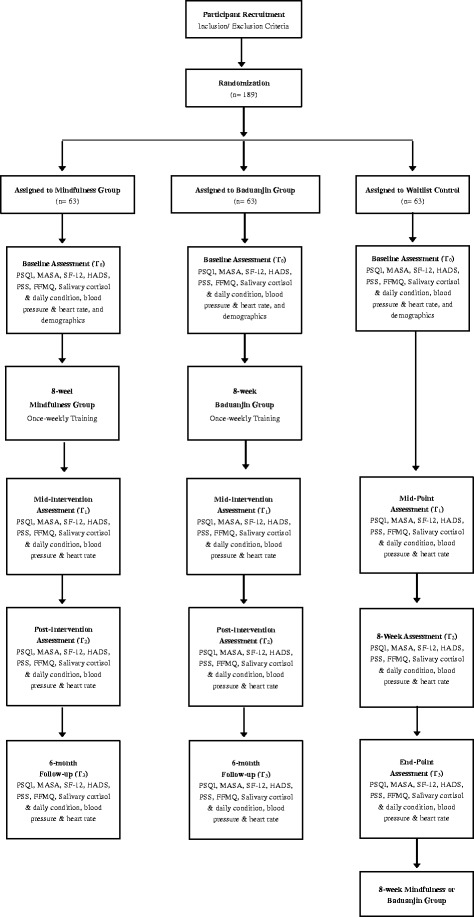
CONSORT diagram of intervention and waitlist control groups and data collection points

Subjects will be recruited via referral from oncologists and from local colorectal cancer support networks by means of electronic mailing lists, printed promotional materials, and newsletters. Figure 1 illustrates the recruitment strategy, treatment received by the intervention group, the waitlist control condition, and the data collection time points of the study.

#### Assignments of interventions

A trained research assistant with a background in psychology or social work will be responsible for enrolling and contacting potential participants over the phone to conduct a screening interview. All eligible participants will be assigned a participant number and will be randomly assigned to one of the study conditions on 1:1:1 ratio using computer-generated random numbers generated by another research assistant on the study team.

### Data collection, management, and analysis

After screening for inclusion and exclusion criteria, participants will be invited to visit the Centre for baseline data collection 1 week before the first group session. Participants from all three arms will be assessed at four time points: at baseline, at the midpoint of the intervention, after the intervention, and at 6-month follow-up.

#### Outcome measures

Assessments will pertain to several areas: (i) cancer-related symptoms and symptom distress, (ii) mental health, (iii) quality of life, (iv) biomarkers, (v) mindfulness, (vi) compliance, and (vii) demographic and clinical details. Self-rated scales and salivary cortisol will be collected from the participants, and blood pressure will be measured by the trained research assistant blinded to the randomization. Measurements will be taken at all time points, with the exception of demographic and clinical details.

#### Cancer-related symptom, symptom distress, and quality of life

- Sleep quality. Quality of sleep will be indexed by the Chinese version of the Pittsburgh Sleep Quality Index (PSQI) [33]. The locally validated Chinese version of the PSQI is a self-report questionnaire that assesses multiple dimensions of sleep over the past month. The 19-item scale generates 7 component scores: subjective sleep quality, sleep latency, sleep duration, habitual sleep efficiency, sleep disturbances, use of sleep medication, and daytime dysfunction. The sum of the seven component scores yields one global score of subjective sleep quality.
- Cancer-related symptom distress. Cancer-related symptom distress is measured with the Chinese version of Memorial Symptom Assessment Scale (MSAS) [34]. The scoring of the 32-item MSAS yields a MSAS Global Distress Index (10 items) to measure overall symptom distress, a Physical Symptom Subscale score (12 items), a Psychological Symptom Subscale score (6 items), and a total MSAS score.
- Health-related quality of life. Health-related quality of life will be assessed with the Chinese (HK) Short form-12 (SF-12) [35]. With 12 items, the scale yields a physical and a mental health summary.
- Anxiety and depressive symptoms. Anxiety and depressive symptoms will be captured by the Cantonese/Chinese version of the Hospital Anxiety and Depression Scale (HADS) [36]. The 14-item scale consists of two subscales (anxiety and depression) of 7 items each, and the items are scored on a 4-point Likert-type scale (0-3).
- Perceived stress. Perceived stress levels will be assessed by the Chinese Perceived Stress Scale (CPSS) [37]. Using a 5-point Likert scale (0-4), the scale consists of 10 items regarding the degree to which life events are appraised as stressful.
- Salivary cortisol. Salivary cortisol collection will be conducted by the participants themselves. Saliva samples will be collected at five prescribed times (upon awakening, 45 min after awakening, 12:00 pm, 5:00 pm, and 9:00 pm) using a collection device, i.e., Salivette, that includes a cotton swab to place under the tongue. Smoking, eating, and drinking should be avoided for 1 h before saliva collection. The samples will be kept frozen at −20 °C. Measures of the participants’ health behaviors and activities on the day of saliva collection will be collected along with the cortisol level, including (i) sleep quantity that day (hours of sleep at night and nap hours), (ii) subjective sleep quality rated on a scale of 1-10, (iii) smoking habit and the number of cigarettes that day, (iv) alcohol/coffee drinking habit and the approximate amount that day, and (v) subjective evaluation of stress levels on the day of collection rated on a scale of 1-10. The preceding measures can affect the diurnal cortisol rhythm and must be considered in our analysis.
- Blood pressure and heart rate. Following recommended procedures by the Canadian Medical Association [38], blood pressure and heart rates will be measured twice on each arm with a 5-min rest intervals between measurements, and the four collected readings will be averaged.
- Mindfulness. The 20-item Chinese Five Facet Mindfulness Questionnaire (Short form) will be used [39]. The scale covers five mindfulness-related domains—observing, describing, acting with awareness, non-judgment of inner experience, and non-reaction to inner experience—on a 5-point Likert scale.
- Compliance. The participants will record their daily duration of qigong or mindfulness meditation practice over the course of the study. This will provide information on the frequency and intensity of practice and the maintenance of self-practice habits after the intervention period.
- Demographics. The participants’ sociodemographic data, such as age, gender, education level, employment status, and marital status, and their clinical profile, including cancer diagnosis, staging, treatment and medication record, onset of mood disturbances if any, critical events around the time of onset, physical exercise habits, complementary treatment used, and psychosocial support service use, will be self-reported.

#### Data management

Data entry will be conducted by a clerical staff trained in research data entry. All participants will be assigned a participant number, and all data will be stored in an onsite server accessible only to the research team members. A range check will be performed for data values.

#### Data analysis

To assess the effectiveness of Baduanjin qigong and mindfulness meditation, analysis of variance and chi-square independence tests will be carried out using SPSS to compare the demographic profiles of the three groups. The study will use latent growth modeling in Mplus 7 to explore the effectiveness of the two mind-body interventions over the four assessment time points, between the respective experimental and waitlist control groups. Traditional analytic techniques such as analysis of variance adopt traditional deletion or substitution methods and may not yield precise estimates in the presence of missing data. Latent growth modeling flexibly analyzes the overall population trajectories using nonlinear modeling such as quadratic growth and the between-person variation in outcome variables, thus allowing analysis of all available data via full information maximum likelihood under missing-at-random assumption. This matches the standard intent-to-treat analytic approach of clinical trials with missing data. The level of statistical significance (p) will be set at 0.05, and *p* values greater than 0.05 but less than 0.10 will be considered to indicate marginal significance. All models will be estimated using the robust maximum likelihood estimator.

To examine the similarities and differential effectiveness of Baduanjin qigong and mindfulness meditation, the treatment effects of the two interventions will be compared directly in multigroup conditional growth models. The linear slope factors of the outcomes will be contrasted across the two intervention groups by computing the difference in the intercepts of the slope factors between the two groups using the model constraint option. The demographic and clinical characteristics that differ significantly across the three groups in the preliminary analysis will be entered as covariates. Influential confounding variables such as the clinical prognosis or the frequency, intensity, and duration of maintenance (compliance) of practice will be controlled.

With baseline data from both the experimental and control groups, correlation analyses will be performed to understand the relationships between the parameters that reflect the mind and body, which include diurnal cortisol patterns, subjective stress, and assessments of psychological symptoms, sleep patterns, and health-related quality of life. Changes in those associations across time can be analyzed by latent growth modeling using Mplus.

The saliva samples will be centrifuged at 3000 rpm for 15 min at room temperature. Cortisol levels will be determined with an enzyme-linked immunosorbent assay kit (Salimetrics, Inc.) in the HKU Clinical Oncology Lab. The assay sensitivity is 0.193 nmol/l, and the intra-assay and inter-assay coefficients of variation are 3 and 10%, respectively. Due to the skewed distribution of salivary cortisol data, a natural logarithm will be used to transform the raw cortisol data to yield an unskewed distribution for analysis. The mean of the five daily cortisol levels will be used, and the total cortisol level will be measured by the area under the curve. To explore the individual trajectories of changes in the cortisol level over time and the complex relationships between different variables, a two-level individual growth curve model using Mplus software will be adopted as cortisol measures at five daily time points are nested within participants. This method is a variant of multiple regression modeling that is appropriate for the nested structure of our data.

## Discussion

Despite the growing family of mind-body practices, few studies juxtaposed their differential outcomes, magnitude and pace of change and post-intervention compliance. Therefore, using a comparative randomized-controlled trial, this study is set out to further establish mind-body practices in psychosocial oncology by exploring the contrasting approaches with which qigong and mindfulness meditation exert their influences. This study will measure both physiological and psychological outcomes which offer a comprehensive understanding on the mental and bodily changes before, during, after each approach and their maintenance. Examining the effects of mind-body practices on circadian cortisol rhythm may shed light on the homeostatic effect of mind-body practices on the dysregulation of the HPA system. Better understanding on culturally popular interventions, in particular, the common and unique effectiveness of different approaches are crucial, as patients and qualified healthcare professionals can select or provide practices which will produce maximum benefits, satisfaction, adherence, and sustainability.
